# O073. Proposal guidelines for epilepsy and headache

**DOI:** 10.1186/1129-2377-16-S1-A193

**Published:** 2015-09-28

**Authors:** Filippo Dainese, Giuliano Avanzini, Angela La Neve, Dario Pruna, Francesco Paladin

**Affiliations:** Epilepsy Center, Neurological Division, Venice, Italy; IRCCS Carlo Besta Foundation Neurological Istitute, Milan, Italy; Epilepsy Center, Neurology Clinic, University of Bari, Bari, Italy; Epilepsy Unit, Child Neuropsychiatry Department, University Hospital, Cagliari, Italy

## Background

Headache and epilepsy are two paroxysmal disorders with epidemiological and clinical comorbidity. Among the primary headaches, migraine, and in particular the subtype with aura (MA) has a significant correlation with epilepsy. Like epilepsy MA is a chronic condition characterized by recurrent focal neurological attacks that are variously accompanied by headache and inconstant autonomic features.

Interictal electroencephalogram (EEG) findings are usually normal although several abnormalities, especially diffuse slowings, have been reported in migraineurs. EEG is not useful in MA with the exception of situations where seizure disorders are to be excluded.

The relationship between headaches and epilepsy is covered in the International Classification of Headache Disorders in chapters 1 and 7 where migraine-triggered seizure (1.4.4), hemicrania epileptica (7.6.1) and post-ictal headache (7.6.2) are collocated. An interictal headache was defined as headache starting not earlier than three hours after a seizure or a headache never proceeding directly into an epileptic fit. Headache caused by an epileptic seizure, occurring during and/or after the seizure and remitting spontaneously within hours or up to 3 days [1, 2]. The classification of the International League against Epilepsy does not refer to this type of disorder. In the last decade other terms for this association have been proposed (epileptic headache or ictal epileptic headache) and some Authors have proposed to abandon or modify some terms (hemicrania epileptica and migralepsy) [3–5].

## Methods

We performed an extensive review of the literature including the terms covered in the ICHD and the terms recently proposed. We rated how the recent literature has influenced the change of the third edition of the ICHD (ICHD 3-*beta*).

## Results and discussion

Despite a high number of papers in this field, no significant changes have been added in the association between headache and epilepsy in the ICHD 3-*beta*. Migralepsy has been confirmed and only shifted by encoding code remaining between the forms for chronic migraine. The term “Hemicrania epileptica” still remains, but the diagnostic criteria have changed whereas this can also be encoded as headache controlateral to the ictal discharge (in the past only ipsilateral). Diagnosis requires the simultaneous onset of headache with EEG-demonstrated ictal discharge. In our opinion, for proper classification of the cases with a strictly temporal association between a headache attack and an epileptic seizure, an ictal EEG is essential for the diagnosis. To our knowledge, it is a feasible guideline proposal shared among the scientific societies that deal with headaches and epilepsy.
